# Polarized Raman Microscopy to Image Microstructure Changes in Silicon Phthalocyanine Thin‐Films

**DOI:** 10.1002/smsc.202300350

**Published:** 2024-03-18

**Authors:** Rosemary R. Cranston, Taylor D. Lanosky, Raluchukwu Ewenike, Sophia Mckillop, Benjamin King, Benoît H. Lessard

**Affiliations:** ^1^ Department of Chemical and Biological Engineering University of Ottawa 161 Louis Pasteur Ottawa K1N 6N5 ON Canada; ^2^ School of Electrical Engineering and Computer Science University of Ottawa 800 King Edward Ave Ottawa K1N 6N5 ON Canada

**Keywords:** organic thin‐film transistors, polymorph, Raman microscopy, silicon phthalocyanine, thermal annealing

## Abstract

The choice of deposition technique and post deposition treatment can significantly influence the performance of organic electronic devices by altering the complex relationship between film properties and charge transport. Herein, the influence of deposition method and post deposition thermal annealing on the thin‐film properties of an emerging semiconductor, bis(tri‐*n*‐propylsilyl oxide) SiPc ((3PS)_2_‐SiPc), is examined by polarized Raman microscopy. Comparing physical vapor deposition (PVD) and spin‐coating, the orientation of (3PS)_2_‐SiPc molecules in films is determined and further characterized by X‐ray diffraction to assess variations in microstructure and morphology due to thermal annealing. Despite differences in film formation, non‐annealed organic thin‐film transistors (OTFTs) fabricated by PVD and spin‐coating resulted in similar electron mobilities (*μ*_*e*_) on the order of 10^−2^ cm^2^ V^−1^ s^−1^ and threshold voltages (*V*_*T*_) of 10–20 V. Films fabricated by PVD annealed at 175 °C transition to a new polymorphic form with molecules aligned at a higher angle to the substrate and exhibiting reduced device performance. Conversely, spin‐coated films do not undergo any new polymorph formation or structural reorganization with thermal annealing. PVD fabricated films are thus more readily able to undergo transformations to structure and morphology with post deposition processing, while the microstructure of spin‐coated films is established at the time of deposition.

## Graphical abstract

Through spin‐coating and physical vapor deposition, the influence of fabrication method and post deposition processing on the microstructure of organic small molecule thin‐films is assessed by polarized Raman microscopy. Changes in film structure are correlated to the semiconducting properties of organic thin‐film transistors, highlighting the crucial role of deposition method in dictating film morphology and device performance.

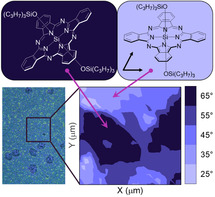

## 1 Introduction

The micro‐ and nanoscale features of organic thin‐films have a significant impact on resulting device performance.^[^ ^1^, ^2^, ^3^, ^4^ ^]^ While topological imaging can be used to study film homogeneity and texture^[^ ^5^, ^6^ ^]^ and diffraction techniques can assay film crystallinity and molecular orientation,^[^ ^7^, ^8^ ^]^ Raman microscopy offers the unique ability to simultaneously characterize both film morphology and structural orientation while maintaining high spatial resolution. Material characterization by Raman microscopy involves the measurement and identification of distinct vibrational modes and the analysis of their relative intensities across a sample area, ultimately providing compositional maps. The intensity of a Raman band depends on the orientation of a materials’ axes of symmetry with respect to the direction of excited laser and scattered light polarizations.^[^ ^9^, ^10^ ^]^ Thus, Raman microscopy enables the nondestructive characterization of a wide range of samples with no restrictions on substrate material, dimension, or specialized sample preparation. Additionally, unlike diffraction methods which require highly crystalline samples, Raman microscopy can be used to gather information on amorphous and crystalline phases while being faster, more accessible, and less energy‐intensive than synchrotron methods.

**Figure 1:**
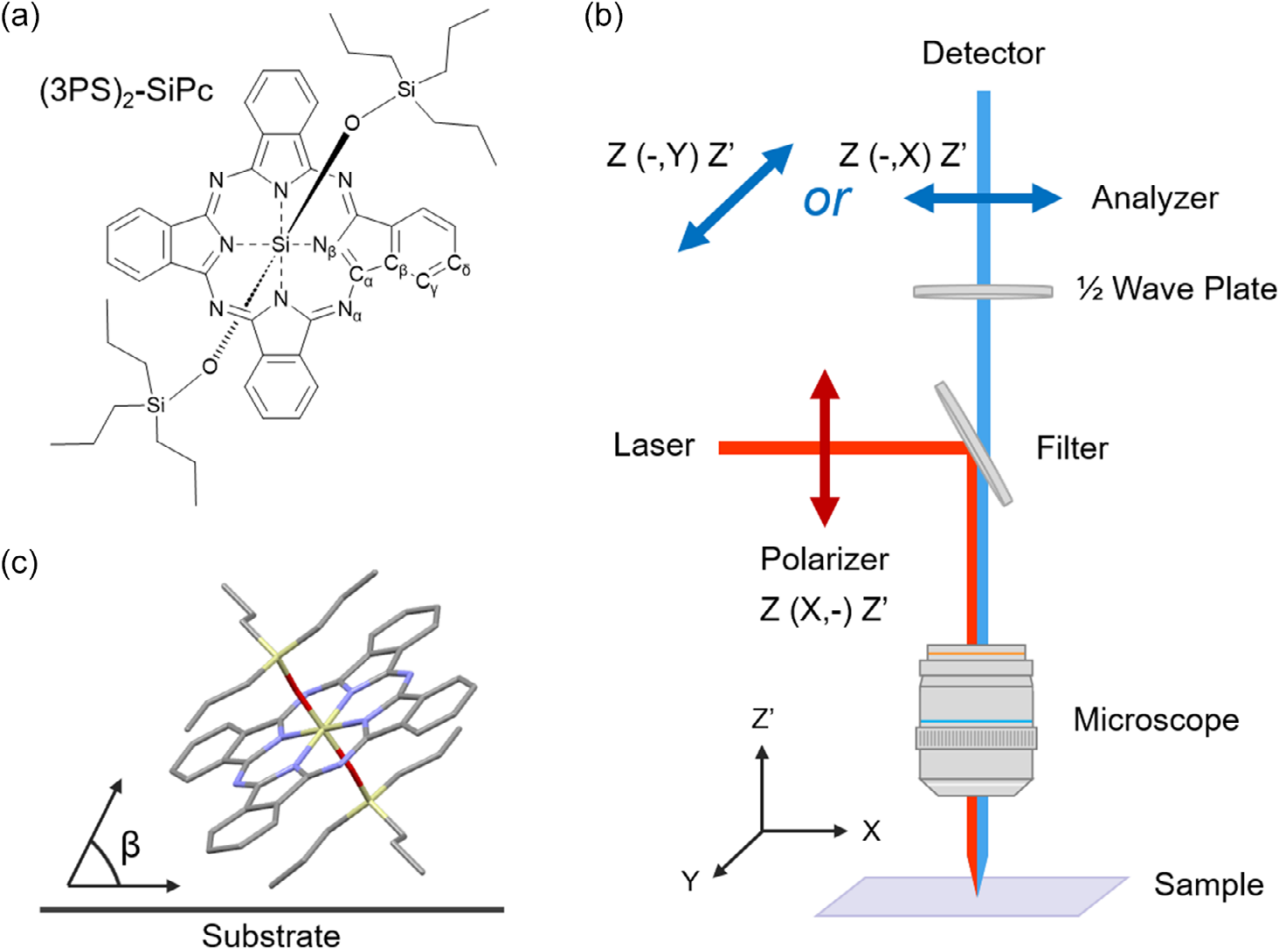
a) Structure and atomic notation of the (3PS)_2_‐SiPc molecule. b) Diagram of the polarized Raman microscope configuration and the orientation of polarization axes with respect to the sample and the corresponding Porto notation. c) Diagram representing the *β* of a (3PS)_2_‐SiPc molecule to the substrate.

Polarized Raman microscopy can be used to quantify the orientation of an organic thin‐film either by the external crystal modes, if the crystal structure of the material is known, or by the internal molecular vibration approach.^[^ ^11^, ^12^, ^13^, ^14^ ^]^ The internal molecular vibration approach has been extensively used to determine the molecular orientation, packing structure, and polymorph formation of planar and nonplanar small molecule thin‐films including copper, zinc, and aluminum chloride phthalocyanines (Pcs).^[^ ^13^, ^15^, ^16^, ^17^, ^18^, ^19^, ^20^ ^]^ However, to the best of the authors’ knowledge, this method has yet to be used for the analysis of tetravalent metal and metalloid Pcs such as silicon phthalocyanines (R_2_‐SiPcs). As promising semiconducting materials, R_2_‐SiPcs have well‐established syntheses with a customizable molecular design and a wide range of processing compatibilities,^[^ ^21^, ^22^, ^23^ ^]^ enabling their use as the active material in organic photovoltaics,^[^ ^24^, ^25^ ^]^ thin‐film gas sensors,^[^ ^26^ ^]^ and organic thin‐film transistors (OTFTs).^[^ ^27^, ^28^ ^]^ In particular, bis(tri‐*n*‐propylsilyl oxide) SiPc ((3PS)_2_‐SiPc, **Figure** **1**a) is a high‐performing n‐type R_2_‐SiPc derivative which is processable by both sublimation and solution deposition techniques.^[^ ^29^ ^]^ As thin‐film formation, morphology, packing, and trap density play critical roles in the effectiveness and utility of a material for device applications,^[^ ^30^, ^31^, ^32^, ^33^ ^]^ understanding the interplay between film fabrication and microstructural properties on device performance is paramount for the further development and advancement of n‐type devices based on this family of organic semiconductors.

In this work, we report the first use of polarized Raman surface maps to estimate the molecular angle of (3PS)_2_‐SiPc films by the internal molecular vibration approach. From single‐crystal X‐ray diffraction (XRD) and UV–vis spectroscopy, (3PS)_2_‐SiPc is determined to be a planar molecule with *D*_*4h*_ symmetry and a slip‐stacked packing structure,^[^ ^29^, ^34^ ^]^ enabling characterization by the internal molecular vibration approach and polarized Raman microscopy. Thin‐films of (3PS)_2_‐SiPc were fabricated by physical vapor deposition (PVD) and spin‐coating, and characterized by thin‐film XRD, and polarized Raman microscopy to assay film crystallinity, morphology, and molecular orientation. Differences in fabrication method were correlated to OTFT device performance by comparing electron field‐effect mobilities (*μ*_*e*_), hysteresis, and threshold voltages (*V*_*T*_), while the influence of post deposition thermal annealing on film microstructure and polymorph formation was investigated by in situ polarized Raman surface maps of films fabricated by PVD. Key annealing temperatures were identified ex situ for (3PS)_2_‐SiPc thin‐films fabricated by both sublimation and solution deposition methods, with considerable differences in the reorganization of PVD and spin‐coated films observed at high‐temperature annealing. Here, we find that the initial differences in film microstructure and morphology resulting from the choice of material deposition dictate the final film microstructure post thermal treatment. This is in opposition to the more intuitive use of high‐temperature annealing as a method to rectify film properties post deposition.

## 2 Results and Discussion

### 2.1 Thin‐Film Fabrication

As the extent of Raman scattering for a given mode is described by its Raman tensor, the interaction between molecules and the polarization direction of light can be related to Raman intensity.^[^ ^9^, ^10^ ^]^ From the internal molecular vibration approach, information on the orientation and distribution of molecules in a film is obtained from the elements of the scattering tensor matrix for a selected vibration.^[^ ^9^, ^10^ ^]^ The components of a Raman tensor are probed by quantifying the intensity ratio of diagonal (parallel or *Z*(*X*,*X*)*Z*′) and nondiagonal (cross or *Z*(*X*,*Y*)*Z*′) polarization of incident and scattered light (Figure 1b). For Pcs with *D*_*4h*_ symmetry, the Raman‐active nondegenerate modes A_1g_ and B_1g_ are in‐plane vibrations corresponding to molecular symmetry, and can therefore be used to determine film orientation by polarized Raman spectroscopy.^[^ ^11^, ^12^, ^15^, ^16^ ^]^ In the case of planar Pc thin‐films, the intensity of a Raman band at *Z*(*X*,*X*)*Z*′ and *Z*(*X*,*Y*)*Z*′ polarizations can be described by Equation (1) and (2) found in the Experimental Section.^[^ ^11^, ^12^, ^15^, ^16^ ^]^ The orientation of a molecule in a film can then be estimated using Equation (3) to calculate the angle of molecules relative to the substrate (*β*, Figure 1c) using the ratio of Raman band intensities at each polarization.

**Figure 2:**
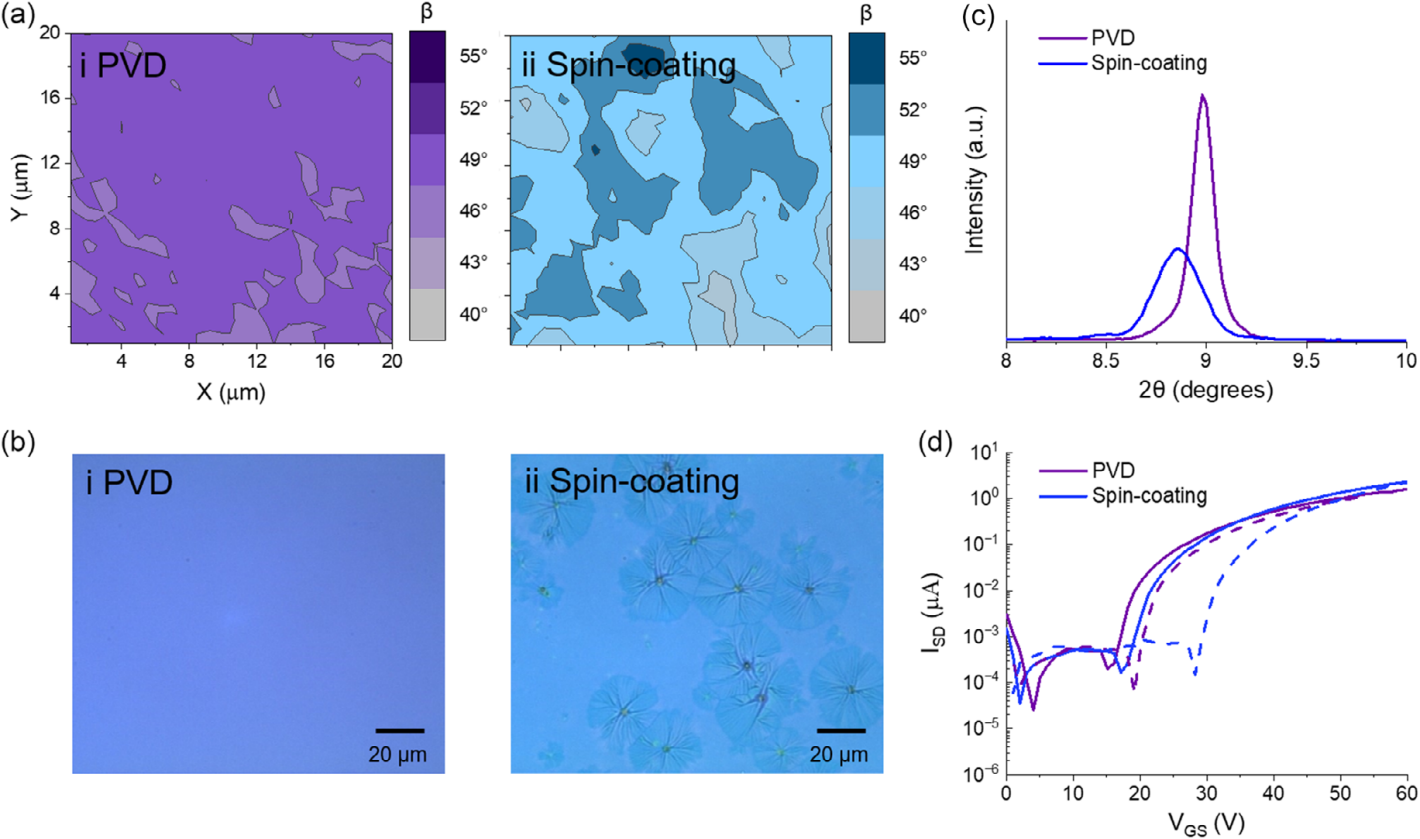
a) Maps of *β* (20 × 20 μm) between (3PS)_2_‐SiPc and substrate estimated from polarized Raman spectra, b) bright‐field real‐color microscopy images, c) XRD patterns, and d) characteristic forward (solid line) and reverse (dashed line) transfer curves of (3PS)_2_‐SiPc OTFTs and thin‐films fabricated by (i) PVD and (ii) spin‐coating. No post deposition thermal annealing was performed on thin‐films in this dataset.

Through Raman surface maps taken with *Z*(*X*,*X*)*Z*′ and *Z*(*X*,*Y*)*Z*′ polarizations, the integral intensity ratio of the B_1g_ pyrrole stretch mode was found, and the corresponding *β* of (3PS)_2_‐SiPc molecules to the substrate estimated for films fabricated by PVD (150 nm) and spin‐coating (95 nm). For films fabricated by PVD, **Figure** **2**ai shows a uniform surface with (3PS)_2_‐SiPc orientated predominantly pseudo‐edge‐on, with an average *β* of approximately 52° to the substrate, which agrees with the previous work by scanning transmission X‐ray microscopy.^[^ ^29^ ^]^ Conversely, spin‐coated films show a more varied orientation (Figure 2aii) with much of the film aligned pseudo‐edge‐on to the substrate with a *β* between 46° and 49°. However, there exhibits areas with lower (*β*_*min*_ = 44°) and higher (*β*_*max*_ = 57°) angles to the substrate compared to films fabricated by PVD (*β*_*min*_ = 47° and *β*_*max*_ = 55°). The increased inhomogeneity of spin‐coated films is reflected in the microscopy images where PVD yielded films with an effectively uniform surface morphology; spin‐coated films were marked by large spherulites roughly 35 μm across (Figure 2b).

XRD patterns of PVD and spin‐coated films shown in Figure 2c indicate a difference in film crystallinity between the two fabrication methods. Films deposited by PVD exhibit a narrow high intensity peak at 2*θ* = 9.02° with a full width at half maximum (FWHM) of 0.13°, while spin‐coated films exhibit a relatively broad peak with reduced intensity at 2*θ* = 8.86° and a FWHM of 0.26°. The higher peak intensity of films fabricated by PVD may partly be due to a larger film thickness compared to spin‐coated; however, the narrower peak shape and shift in 2*θ* indicate a film with larger crystallite domains and a smaller intermolecular *d*‐spacing. The broad low intensity XRD peak observed in spin‐coated films agrees with the larger distribution and variation in *β* seen by polarized Raman surface maps. Additionally, analogous to previous work,^[^ ^29^ ^]^ neither fabrication method results in an XRD pattern that matches the polymorphic forms of (3PS)_2_‐SiPc predicated from single crystal (Figure S1, Supporting Information).

The impact of (3PS)_2_‐SiPc film deposition on the electrical characteristics of OTFTs was determined with characteristic transfer and output curves found in Figure S2 and S3, Supporting Information. OTFTs fabricated by PVD and spin‐coating exhibited notably similar metrics with PVD OTFTs having an average *μ*_*e*_ of 0.44 ± 0.05 × 10^−2^ cm^2^ V^−1^ s^−1^ and *V*_*T*_ of 16.6 ± 1.7 V, and spin‐coated OTFTs having an average *μ*_*e*_ of 0.74 ± 0.17 × 10^−2^ cm^2^ V^−1^ s^−1^ and *V*_*T*_ of 21.0 ± 2.8 V (Table S1, Supporting Information). Between the two fabrication methods, spin‐coating resulted in a higher *V*_*T*_ and increased hysteresis between the forward and reverse transfer curves (Figure 2d). This could be a result of bias stress behavior during device operation causing a shift in *V*_*T*_ over time, which is more prominent in solution processed films due to their increased defect density and trapping sites.^[^ ^35^, ^36^, ^37^, ^38^ ^]^ As the driving force, time scale, and controllability of crystallite nucleation and film growth for PVD and spin‐coating are very different, it is expected that OTFTs fabricated by these two methods would exhibit large differences in device performance. However, our previous work demonstrated how the relationship between crystallinity, grain boundary effects, surface roughness, and molecular orientation has less effect on the performance of (3PS)_2_‐SiPc OTFTs due to its unique material properties.^[^ ^29^ ^]^ This result is corroborated herein as despite differences in film microstructure and morphology between (3PS)_2_‐SiPc films fabricated from sublimation or solution, the electrical characteristics of OTFTs are comparable.

### 2.2 In Situ Thermal Annealing

**Figure 3:**
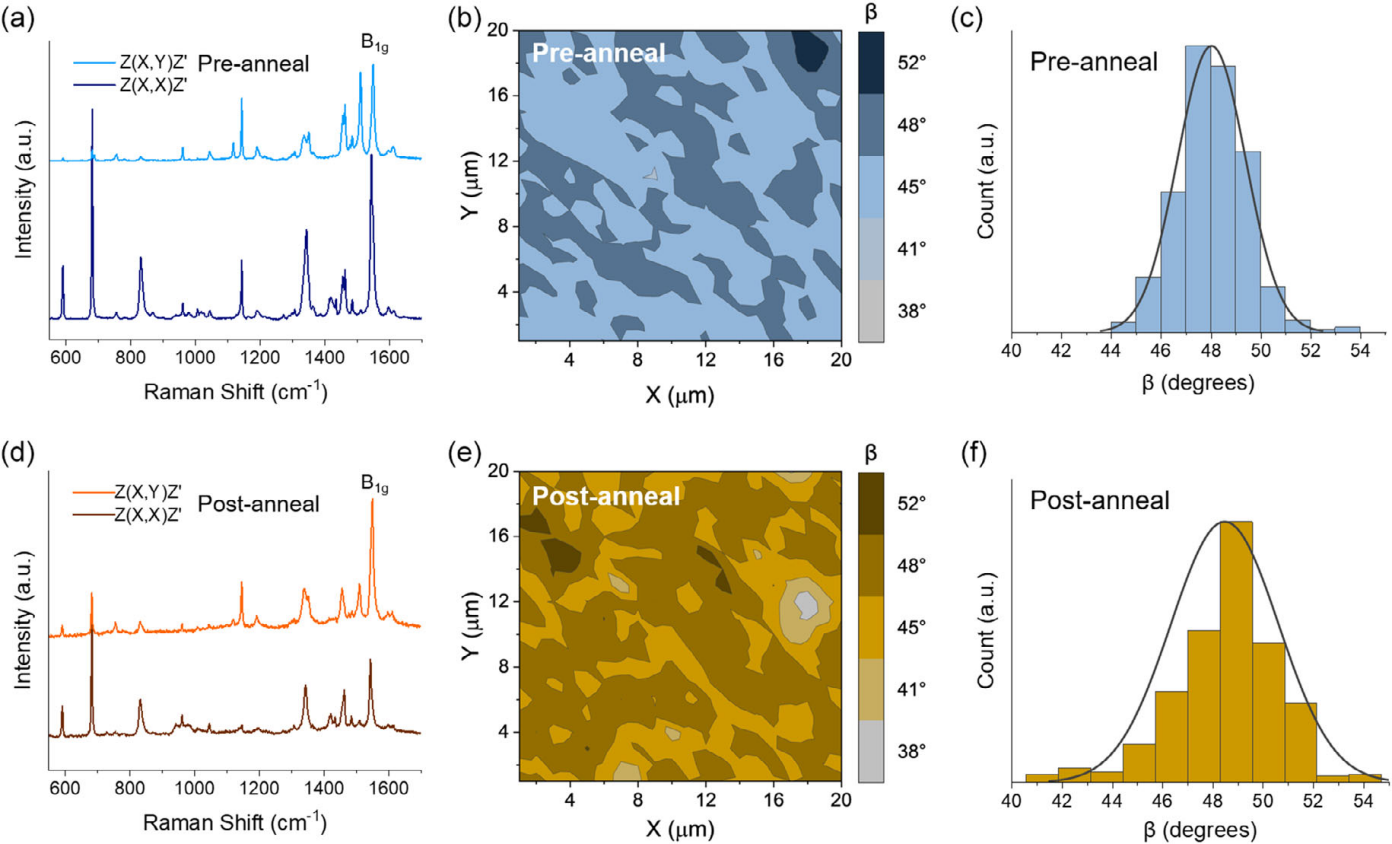
a,d) Polarized Raman spectra, b,e) in situ maps of *β* (20 × 20 μm) between (3PS)_2_‐SiPc and substrate estimated from polarized Raman spectra, and c,f) distribution histograms of *β* shown in Raman maps of (3PS)_2_‐SiPc thin‐films fabricated by PVD pre‐ and post‐annealing. All measurements are taken at the same location on the film.

To ascertain the effects of film reorganization, in situ thermal annealing characterized by Raman microscopy was conducted on thin‐films fabricated by PVD such that pre‐annealed polarized Raman surface maps were taken at a starting temperature of 25 °C and used to estimate *β*. The temperature was then increased at a rate of 5 °C min^−1^ until 180 °C which was held for 30 min before cooling to 25 °C where post‐annealed polarized Raman surface maps were measured at the identical location on the film. **Figure** **3**a,d presents the Raman spectra using *Z*(*X*,*X*)*Z′* and *Z*(*X*,*Y*)*Z′* polarizations pre‐ and post‐annealing at the same location on the film. For *Z*(*X*,*X*)*Z′* polarization, the A_1g_ band (591 cm^−1^) associated with benzene ring deformation, the B_1g_ band (681 cm^−1^) related to macrocycle breathing, and the B_1g_ band (1545 cm^−1^) correlated to pyrrole stretch are clearly present.^[^ ^39^, ^40^, ^41^ ^]^ By changing the polarization to *Z*(*X*,*Y*)*Z′*, a loss in intensity is observed such that the B_1g_ pyrrole stretch band remains most prominent. Distinct changes in the fingerprint region of the spectral range (1350–1550 cm^−1^), which correspond to isoindole stretching, vibrations of the N—Si and C—H bonds, and displacements of the C_α_—N_β_—C_α_ bridge bond of the (3PS)_2_‐SiPc molecule,^[^ ^40^, ^42^, ^43^ ^]^ are noticeable with changing polarization. For directionally orientated films, the scattering intensity for a particular Raman mode depends on the alignment of the bond axes relative to the direction of polarization. Therefore, when the polarization is parallel or perpendicular to a bond axis, the intensity of Raman scattering will not be equal, and information regarding orientation can be determined. Greater C—C and C—N intensity with *Z*(*X*,*X*)*Z*′ (parallel) polarization compared to *Z*(*X*,*Y*)*Z*′ (perpendicular) polarization indicates the (3PS)_2_‐SiPc molecules are more closely aligned with these bond axes parallel to the substrate with a lower *β*. Conversely, more intense B_1g_ scattering with *Z*(*X*,*Y*)*Z*′ polarization relative to *Z*(*X*,*X*)*Z*′ suggests that the (3PS)_2_‐SiPc molecules have a greater angle to the substrate. The pre‐ and post‐annealed polarized Raman spectra in Figure 3a,d reveal that after thermal annealing the intensity of the B_1g_ peak increases with *Z*(*X*,*Y*)*Z*′ polarization, whereas the pre‐annealed film shows a decrease in peak intensity at this polarization. This polarization‐dependent change in B_1g_ peak intensity indicates a transition in the thin‐film structure resulting from thermal annealing.

Figure 3b,e displays maps of *β* between (3PS)_2_‐SiPc and the substrate for pre‐ and post‐annealed thin‐films estimated from polarized Raman surface maps. The pre‐annealed film shows a similar orientation to Figure 2a with a *β* around 48° to the substrate. After thermal annealing more areas with a *β* of 48° are present with additional regions of much lower and higher angle (*β*_*min*_ = 38° and *β*_*max*_ = 54°) that are not found in the pre‐annealed map (Figure 3e). This can also be seen through the histograms presented in Figure 3c,f, where the distribution of *β* for the post‐annealed film is much wider than the pre‐annealed film. As these measurements were conducted in situ to ensure the same film location, the difference in *β* and film uniformity of pre‐ and post‐annealed thin‐films is therefore not a result of deposition or variation in measurement region, but rather a direct change caused by thermal annealing. Post deposition thermal annealing is a common method to alter the microstructure of Pc thin‐films and induce a polymorphic change.^[^ ^29^, ^44^, ^45^ ^]^ As PVD films undergo a microstructural change with thermal annealing, spin‐coated films will likely have a similar orientation change. The film reorganization caused by thermal annealing suggests that a morphological change may also occur, which coupled with a change in *β* would imply a large difference in the electrical performance of OTFTs pre‐ and post‐thermal annealing.

### 2.3 Post Deposition Thermal Annealing Temperature

**Figure 4:**
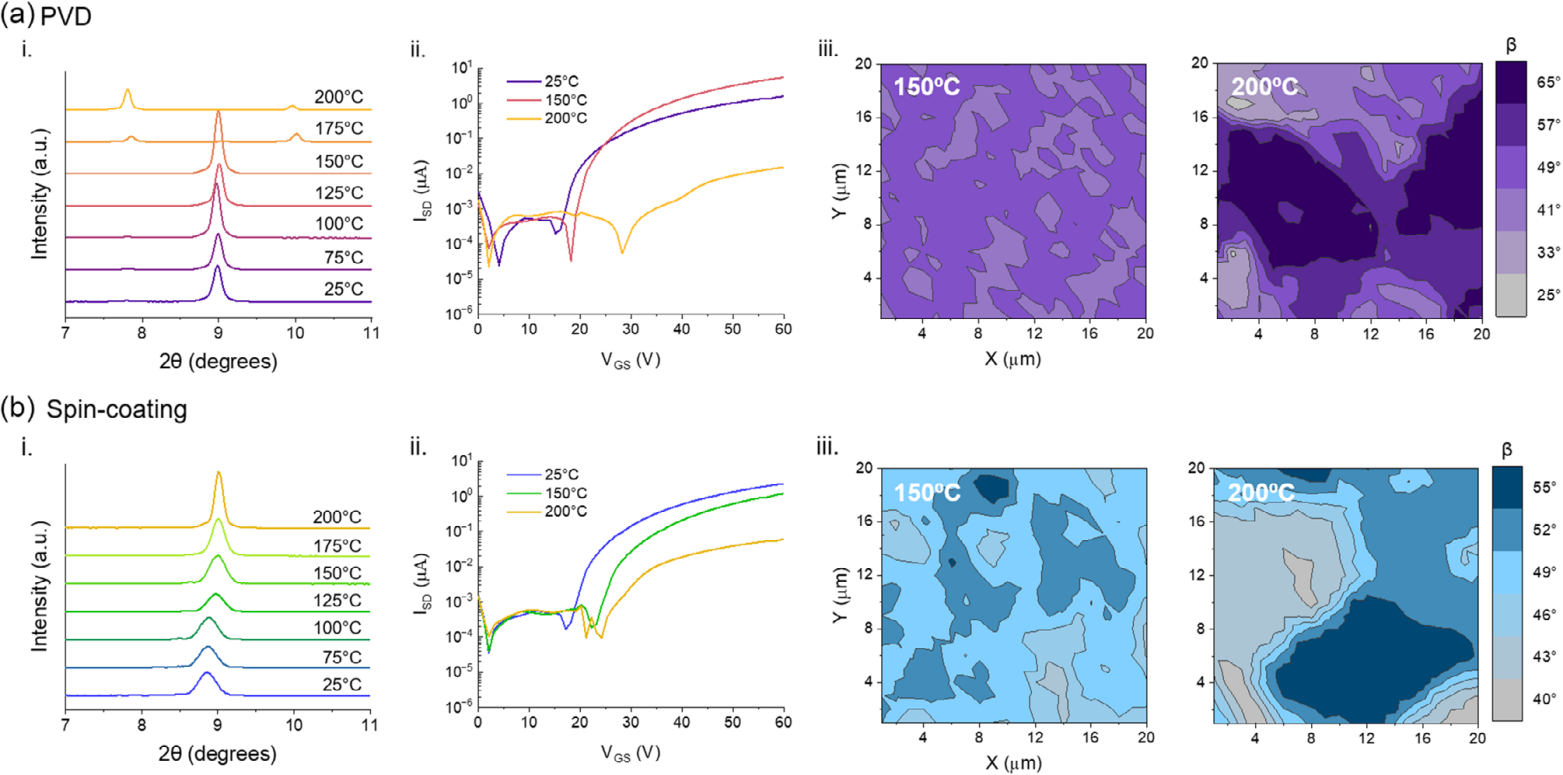
(i) XRD patterns, (ii) characteristic transfer curves, and (iii) maps of *β* (20 × 20 μm) between (3PS)_2_‐SiPc and substrate estimated from polarized Raman spectra of (3PS)_2_‐SiPc thin‐films fabricated by a) PVD and b) spin‐coating thermally annealed at indicated temperatures.

For PVD and spin‐coated films, the post deposition thermal annealing temperature was varied from 25 °C (no thermal annealing) to 200 °C to assay microstructural changes with increasing annealing temperature. A maximum annealing temperature of 200 °C was selected as (3PS)_2_‐SiPc readily sublimes above 200 °C under 100 mTorr pressure.^[^ ^34^ ^]^ The XRD patterns of PVD films, displayed in **Figure** **4**ai, exhibit an increase in peak intensity at 2*θ* ≈ 9.0° with increasing annealing temperature, suggesting higher temperatures yield more crystalline films. After annealing at 175 °C, the films fabricated by PVD exhibit a drastic change in film structure, with a complete loss of the 2*θ* ≈ 9.0° peak and the emergence of two lower intensity peaks at 2*θ* ≈ 7.8° and 2*θ* ≈ 10.0°. The total change in XRD pattern indicates PVD films transition from one polymorphic form to another when annealed at 175 °C and above. Notably spin‐coated films do not undergo the same polymorphic change with annealing temperature as shown in Figure 4bi. For spin‐coated films, an increase in peak intensity and a shift in peak location from 2*θ* = 8.86° to 9.00° is observed with increasing annealing temperature. However, no change in polymorphic form occurs after annealing at 175 °C contrary to the transition observed in films fabricated by PVD. Microscope images of PVD fabricated films show a large difference in surface characteristics only at annealing temperatures of 175 and 200 °C, while spin‐coated films show morphological changes throughout the temperature range (Figure S4, Supporting Information). Although nonpolarized Raman spectroscopy has previously been used to identify polymorph formation in Pc films,^[^ ^46^, ^47^, ^48^ ^]^ no significant change in the nonpolarized Raman spectra or location of key Raman bands (1341, 1458, and 1545 cm^−1^) was observed with annealing temperature as shown in Figure S5 and S6, Supporting Information, respectively.

Similar to in situ measurements, polarized Raman spectra of films fabricated by PVD and spin‐coating exhibit a change in B_1g_ peak intensity ratio with post deposition thermal annealing (Figure S7 and S8, Supporting Information). From Figure 4aiii, the *β* map of a film fabricated by PVD annealed at 150 °C is comparable to that of a non‐annealed film (Figure 2ai), with the majority of (3PS)_2_‐SiPc molecules aligned approximately 49° to the substrate. Annealing at or above 175 °C results in an increase in *β* to 56° and a much larger distribution in angle with a minimum and maximum of 30° and 74° when annealed at 200 °C, compared to 47° and 51° when annealed at 150 °C. In addition to the more varied film and less homogenous microstructure, the increase in *β* agrees with the formation of a new polymorph as identified by XRD. Conversely, spin‐coated films annealed at 200 °C have a comparable average *β* to films annealed at 150 °C, however with more (3PS)_2_‐SiPc molecules aligned 52°–55° to the substrate (Figure 4biii). The minimum, maximum, and average *β* of (3PS)_2_‐SiPc films annealed at each temperature for both deposition methods can be found in Table S2, Supporting Information. In addition to annealing temperature, annealing times of 15, 30, and 60 min were studied at a constant temperature, with no significant changes in PVD or spin‐coated film microstructure observed by XRD and polarized Raman surface maps (Figure S9, Supporting Information). The transition of PVD fabricated films to a new polymorph is therefore primarily brought on by thermal annealing temperature, while spin‐coated films do not undergo polymorph formation with annealing temperature, but rather only exhibit changes in film crystallinity and homogeneity.

Characteristic transfer curves of PVD and spin‐coated OTFTs non‐thermally annealed, annealed before the polymorph transition temperature at 150 °C, and annealed after the polymorph transition temperature at 200 °C are shown in Figure 4aii, bii. For OTFTs fabricated using PVD, thermal annealing at 150 °C resulted in a higher *μ*_*e*_ compared to non‐annealed transistors (Table S1, Supporting Information). This can be attributed to the higher degree of crystallinity that is achieved with thermal annealing as determined by XRD. After the polymorph transition temperature, OTFTs annealed at 200 °C experience a severe reduction in device performance, with three orders of magnitude decrease in average *μ*_*e*_ from 1.13 ± 0.13 × 10^−2^ cm^2^ V^−1^ s^−1^ to 7.93 ± 5.7 × 10^−5^ cm^2^ V^−1^ s^−1^. The worse electrical performance of devices annealed at 200 °C could be due in part to the more irregular films that occur with high‐temperature annealing, or a result of the second polymorph of (3PS)_2_‐SiPc which may be less effective at charge transport. The effectiveness of different polymorphs of a material at charge transport depends on the tilt angle between molecular planes and the degree of π‐electron overlap, with higher conductivity observed by polymorphs with increased π‐electron overlap and multidimensional electronic coupling.^[^ ^29^, ^49^, ^50^ ^]^ Therefore, the secondary polymorph of (3PS)_2_‐SiPc likely results in reduced device performance due to decreased π‐electron overlap as a result of the solid‐state arrangement of molecules in thin‐films fabricated by PVD. Spin‐coated films experience a similar reduction in device performance with average *μ*_*e*_ decreasing from 0.55 ± 0.15 × 10^−2^ cm^2^ V^−1^ s^−1^ to 2.09 ± 2.0 × 10^−4^ cm^2^ V^−1^ s^−1^ when annealed at 150 versus 200 °C. However, spin‐coated OTFTs exhibit less of a decrease in device performance compared to OTFTs fabricated by PVD, likely due to film inhomogeneity rather than microstructural reorganization such as polymorph formation.

All thin‐films and OTFTs were fabricated using the same substrate, surface modification, active material, and device architecture, with the only difference in fabrication being deposition method. As the driving force of crystallite nucleation and thin‐film growth for PVD and spin‐coating are quite different, it is expected that films fabricated by these methods will have different properties and characteristics. Pc molecules can be considerably mobile within a film and have been shown to reorientate into more energetically favorable positions with annealing temperature,^[^ ^51^, ^52^ ^]^ solvent exposure,^[^ ^53^, ^54^ ^]^ and even strong magnetic and gravitational fields.^[^ ^55^, ^56^, ^57^ ^]^ For the films fabricated herein, after applying sufficient thermal energy to induce reorganization the resultant microstructures of films deposited by PVD and spin‐coating remain unalike, undergoing different processes with high‐temperature annealing. These results suggest that the initial differences in film microstructure and morphology obtained by sublimation or solution processing are too great to overcome by post deposition processes despite the restructuring ability of Pcs. PVD fabricated films can therefore more easily undergo transformations to structure and morphology after deposition, while the molecular arrangement of spin‐coated films is largely determined by the initial deposition process. The greater reorganizational abilities of PVD films may be due in part to the inherent process of a material going from a vapor phase to a solid film. The beginning stages of film formation involve the diffusion of the initial low‐density distribution of molecules across the substrate to thermodynamically stable locations.^[^ ^58^, ^59^, ^60^ ^]^ Meanwhile, for solution processing the rapid evaporation of solvent leaves little time for surface mobility or diffusion resulting in molecules locked in place on the substrate.^[^ ^61^, ^62^, ^63^, ^64^ ^]^ This key difference in the initial stages of film formation between sublimation and solution fabrication may make films deposited by PVD more susceptible to reorganization by post deposition processes.

## 3 Conclusion

Polarized Raman microscopy was used to estimate the molecular orientation of (3PS)_2_‐SiPc thin‐films fabricated by PVD and spin‐coating to determine differences in film microstructure as a result of deposition method and post deposition thermally annealing. Films fabricated by PVD yielded highly uniform, well‐oriented thin‐films while spin‐coating resulted in inhomogeneous films with large variations in morphology and molecular orientation. Although both deposition methods resulted in different film formations, OTFTs fabricated by PVD and spin‐coating exhibited similar *μ*_*e*_ and *V*_*T*_, with the only significant difference being improved hysteresis for OTFTs processed by sublimation. Through XRD and Raman microscopy, thermal annealing was found to have a distinct effect on films fabricated by PVD which was markedly different from spin‐coated films. Films deposited by PVD annealed at 175 °C underwent a polymorphic change in packing structure, characterized by a different diffraction pattern, an increase in *β*, and a reduction in OTFT performance. Conversely, films deposited by spin‐coating exhibited increased crystallinity and *β*, however to a lesser extent than PVD fabricated films, with no observed change in molecular packing or polymorph formation after annealing at or above 175 °C. This work demonstrates the importance of material deposition on thin‐film properties and the impact on device application despite the use of post deposition processing that is primarily used to enhance film formation and device performance.

## 4 Experimental Section

4.1

4.1.1

##### Materials

Bis(tri‐*n*‐propylsilyl oxide) silicon phthalocyanine ((3PS)_2_‐SiPc) was synthesized as described in the literature and purified by train sublimation before use.^[^ ^29^, ^34^ ^]^ Hexamethyldisilane (HMDS, 98+%) was purchased from Thermo Fischer Scientific. Solvents were purchased from commercial suppliers and used as received.

##### OTFT and Thin‐Film Fabrication

Bottom‐gate top‐contact OTFTs were fabricated on 15 mm × 20 mm n‐doped silicon substrates purchased from Ossila with a 300 nm thick thermally grown SiO_2_ dielectric layer. Substrates were cleaned by sequential sonication baths of soapy water, distilled water, acetone, and methanol, and then dried with nitrogen and treated with air plasma for 15 min. HMDS surface treatment was carried out in a nitrogen‐filled glove box by spin‐coating 50 μL of HMDS onto cleaned substrates at 3000 RPM for 30 s, and then dried at 150 °C for 1 h under vacuum. A solution of (3PS)_2_‐SiPc at a concentration of 10 mg mL^−1^ in toluene was prepared by heating at 50 °C for 45 min, and then filtered through a polytetrafluoroethylene membrane with a pore size of 0.45 μm. Solution fabricated thin‐films were fabricated by spin‐coating 60 μL of solution at 1500 RPM for 90 s. PVD fabricated thin‐films were deposited using an Angstrom EvoVac thermal evaporator (*P* < 2 × 10^−6^ torr) at a rate of 0.2 Å s^−1^ until a final thickness of 150 nm was reached. All films were then thermally annealed at indicated temperatures for 30 min. Source–drain electrodes were fabricated by PVD using shadow masks purchased from Ossila (channel length of 30 μm, channel width of 1000 μm) by depositing 10 nm of manganese at a rate of 0.5 Å s^−1^ followed by 50 nm of silver at a rate of 1 Å s^−1^.

##### OTFT Characterization

OTFT characterization was performed using the same procedure outlined in our previous works.^[^ ^65^ ^]^ Characterization occurred in a nitrogen‐filled glove box at room temperature. A Keithley 2614B and a MCC USB DAQ were used to control the source–drain voltage (*V*_*SD*_ = 50 V) and gate voltage (0 V < *V*_*GS*_ < 60 V) to obtain source–drain current (*I*_*SD*_) measurements. The electron field‐effect mobility (*μ*_*e*_) and threshold voltage (*V*_*T*_) were calculated by the MOSFET model.^[^ ^66^ ^]^

##### XRD

XRD measurements were obtained using a Rigaku Ultima IV powder diffractometer with a Cu Kα (*λ* = 1.5418 Å) source, a scan range of 3° < 2*θ* < 12°, and rate of 0.5° min^−1^.

##### Raman Microscopy

Nonpolarized and two types of polarized, *Z*(*X*,*X*)*Z*′ and *Z*(*X*,*Y*)*Z*′, Raman spectra were recorded in the backscattering geometry using a Renishaw inVia InSpec confocal Raman microscope with a custom Linkam stage for in situ temperature control. The Raman microscope uses a Leica Microsystems bright‐field microscope with a DM2700 light source. A 500 mW 532 nm wavelength laser with a 2400 L mm^−1^ grating was used to obtain measurements in the spectral range of 550–1700 cm^−1^, focused on the sample by an X50L objective with a numerical aperture of 0.5. With the objective and laser combination used herein, the Raman microscope has a spectral resolution of 0.3 cm^−1^ (FWHM), a theoretical spatial resolution of approximately 640 nm, and a theoretical depth of focus of approximately 3.0 μm. Calibration was performed prior to all measurements against the 520 cm^−1^ silicon reference peak within 0.5 cm^−1^.

Each polarized Raman spectra was taken at the same location on the sample. Single Raman spectra were taken with 5% laser power (25 mW) and an exposure time of 10 s. The Raman spectra of a clean SiO_2_ substrate can be found in Figure S10, Supporting Information, for reference. Raman maps (20 × 20 μm) were generated from 400 individual spectra using a 1.0 μm step size with a 5% laser (25 mW) power and a 2 s exposure time. Each spectrum was fitted to the theoretical Lorentz curve using Wire 5.6 inVia software to obtain integral intensity (*I*_*XX*_ and *I*_*XY*_) of the B_1g_ pyrrole stretch Raman mode. For planar Pc thin‐films, the intensity of a Raman band at *Z*(*X*,*X*)*Z*′ and *Z*(*X*,*Y*)*Z*′ polarizations is described by Equation 1 and Equation (2).^[^ ^11^, ^12^, ^15^, ^16^ ^]^ Equation (3) can then be used to estimate the angle of (3PS)_2_‐SiPc molecules to the substrate (*β*).

(1) $$I_{XX}=k^{2}{\text{cos}}^{4}\beta$$

(2) $$I_{XY}=\frac{k^{2}}{2}{\text{cos}}^{2}\beta {\text{sin}}^{2}\beta$$

(3) $$\frac{I_{XX}}{I_{XY}}=2{\text{cot}}^{2}\beta$$

in situ Raman maps were obtained by heating the sample at a rate of 5 °C min^−1^ to a starting temperature of 25 °C which was held for 30 min before the spectra were measured with *Z*(*X*,*X*)*Z*′ and *Z*(*X*,*Y*)*Z*′ polarization. The sample was then heated at the same rate to 150 °C and held at temperature for 30 min before cooling at a rate of 5 °C min^−1^ to 25 °C which was held for 10 min before measuring the spectra using both polarizations. This procedure was repeated for a high‐temperature annealing at 180 °C before final cooling to room temperature.

To guarantee no degradation occurred during repeated polarized measurements and mapping experiments, the exposure time, scan repetitions, and mapping step size were carefully controlled. Figure S11, Supporting Information, shows no damage to the sample when the exposure time to the laser was increased from 1 to 10 s for either polarization directions. To generate maps of *β*, it is necessary to measure the mapping area twice, one for each polarization, for a total of 800 scans per *β* map over the same 20 × 20 μm sample area. To ensure that this does not cause any damage or unwanted changes to the film, the spectra and B_1g_ peak intensity at the start and end of the scan were compared for three sequential mapping measurements at each polarization totaling 2400 scans over the same sample area (Figure S12 and S13, Supporting Information). For each polarization, minimal changes in peak intensity and signal‐to‐noise ratio were observed between the start and end of each map and between map repetitions indicating no substantial changes to the film caused by repeated exposure to the laser. Additionally, the mapping step size was decreased from 1.0 to 0.5 μm, resulting in each map consisting of 1600 scans and greatly increasing the amount of time the laser is open to the sample. Figure S14, Supporting Information, displays the effects of lower step size on B_1g_ peak intensity and spectra at the start and end of the map scan. A complete loss in B_1g_ peak intensity and a large increase in the signal‐to‐noise ratio were observed as the scan progressed, indicating film damage over time. Resultantly, all mapping experiments were conducted with a scan step size of 1.0 μm to reduce damage to the sample.

## Conflict of Interest

The authors declare no conflict of interest.

## Supporting information

Supplementary Material

## Data Availability

The data that support the findings of this study are available from the corresponding author upon reasonable request.
