# Supplementary material for: Polarized Raman Microscopy to Image Microstructure Changes in Silicon Phthalocyanine Thin‐Films
Source: Small Sci. 2024 Mar 18;4(6):2300350. doi: 10.1002/smsc.202300350 (PMC11935273; doi:10.1002/smsc.202300350)
Supplement: Supplementary file 1 — Supplementary Material [file SMSC-4-2300350-s001.pdf]

Supporting Information

Polarized Raman Microscopy to Image Microstructure Changes in Silicon Phthalocyanine Thin-Films

Rosemary R. Cranston, Taylor D. Lanosky, Raluchukwu Ewenike, Sophia Mckillop, Benjamin King, and Benoît H. Lessard[*]

R. R. Cranston, T. D. Lanosky, R. Ewenike, S. Mckillop, B. King, B.H. Lessard

University of Ottawa, Department of Chemical and Biological Engineering, 161 Louis Pasteur, Ottawa, ON, Canada

B.H. Lessard

University of Ottawa, School of Electrical Engineering and Computer Science, 800 King Edward Ave, Ottawa, ON, Canada

E-mail: benoit.lessard@uottawa.ca

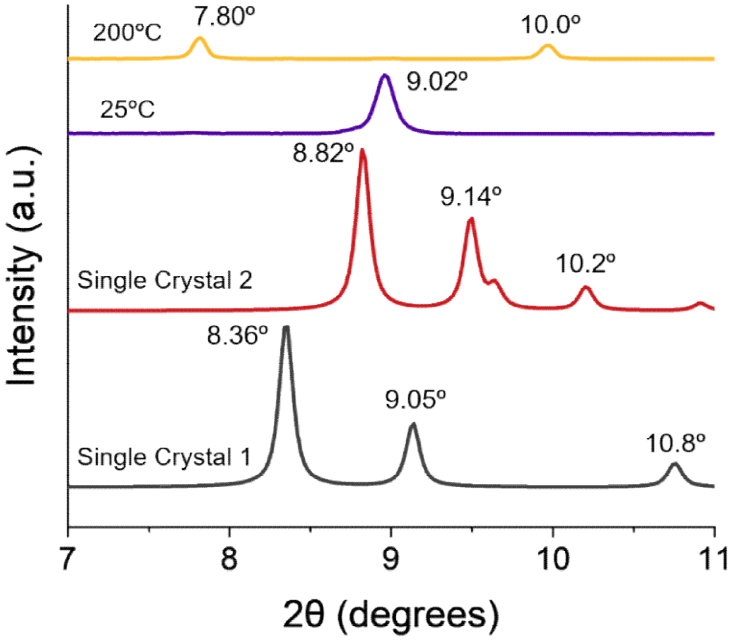

Figure S1. XRD patterns of $(3PS)_2$-SiPc thin-films fabricated by PVD thermally annealed at 25ºC and 200ºC, and XRD pattern of polymorph 1 and polymorph 2 predicated from single crystal data using Mercury: visualization and analysis of crystal structures, from the Cambridge Crystallographic Data Centre (CCDC# 2091746 and CCDC# 2067659).

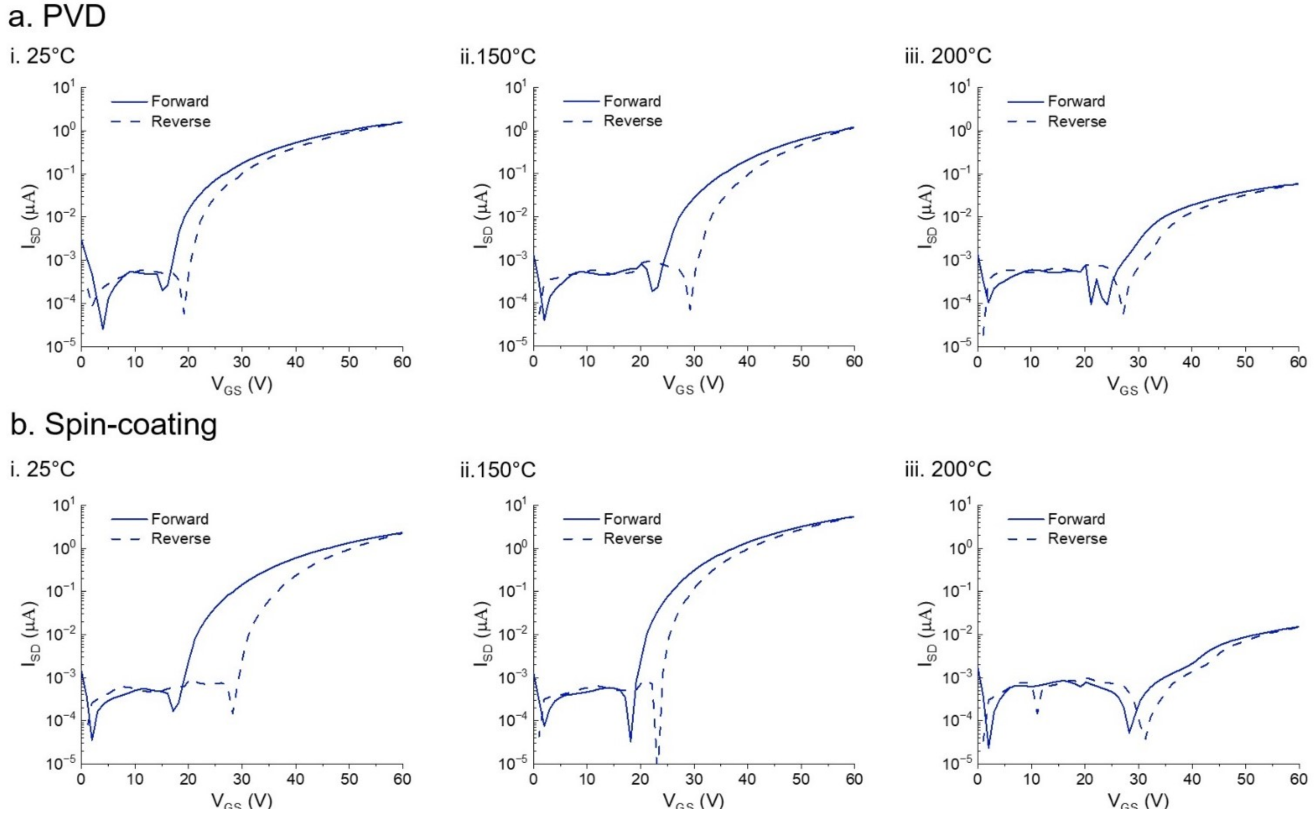

Figure S2. Characteristic forward and reverse transfer curves of (a) PVD and (b) spin-coating fabricated $(3PS)_2$-SiPc OTFTs annealed at (i) 25°C, (ii) 150°C and (iii) 200°C characterized at room temperature in a nitrogen environment.

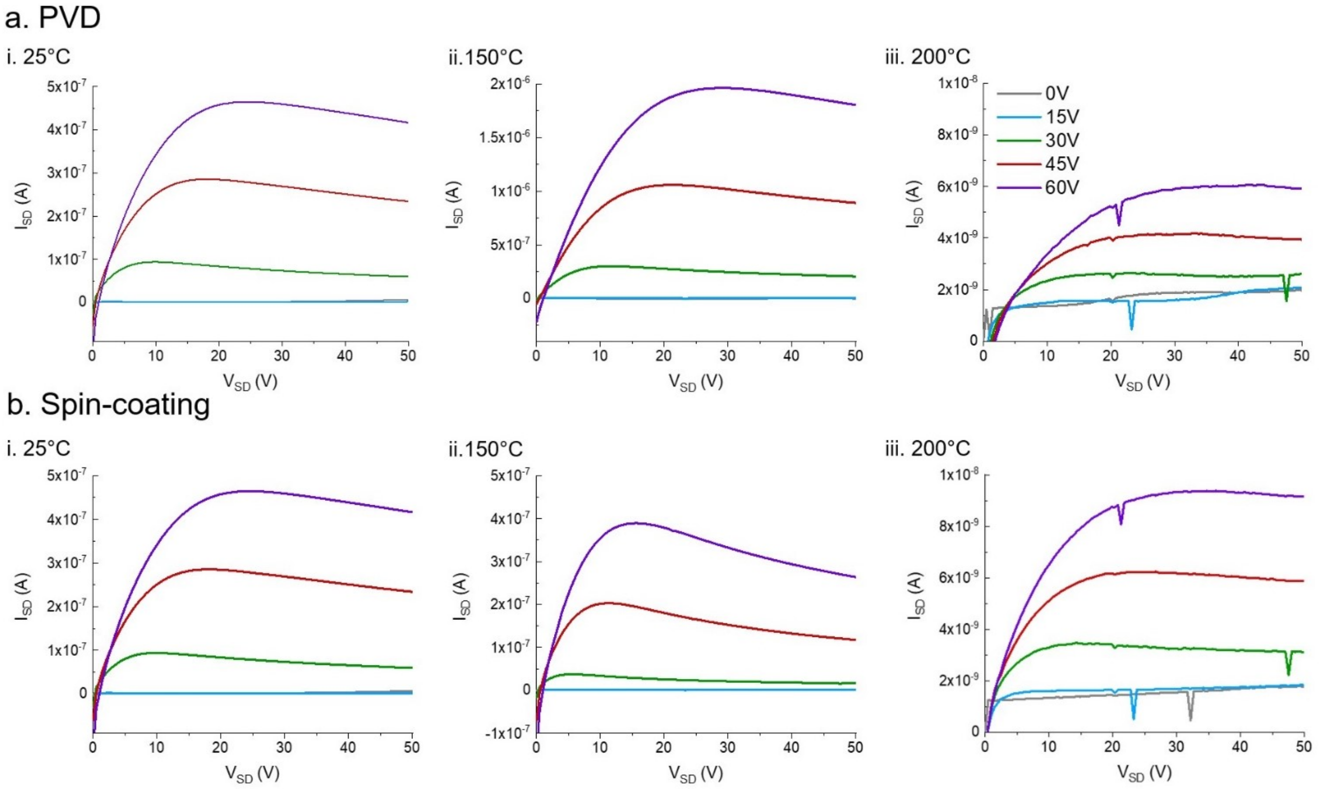

Figure S3. Characteristic output curves of (a) PVD and (b) spin-coating fabricated $(3PS)_2$-SiPc OTFTs annealed at (i) 25°C, (ii) 150°C and (iii) 200°C characterized at room temperature in a nitrogen environment. All OTFTs characterized at gate voltages of 0 V (grey), 15 V (blue), 30 V (green), 45 V (red), and 60 V (purple).

Table S1. Average electrical characteristics of OTFTs calculated from *n* transistors.

**Deposition**	**Temp.**	**$\mu_{e, avg}$(x 10^{-2} cm^2 V^{-1} s^{-1})**	**V_T (V)**	**$I_{on/off}$**	***n***
PVD	25°C	0.44 ± 0.05	16.6 ± 1.7	10^5	37
	150°C	1.13 ± 0.13	21.5 ± 1.2	10^5	40
	200°C	7.93 ± 5.7 x 10^{-3}	24.4 ± 2.1	10^3	30
Spin Coat	25°C	0.74 ± 0.17	21.0 ± 2.8	10^5	39
	150°C	0.55 ± 0.15	25.9 ± 3.1	10^4	33
	200°C	2.09 ± 2.0 x 10^{-2}	23.6 ± 4.2	10^3	8

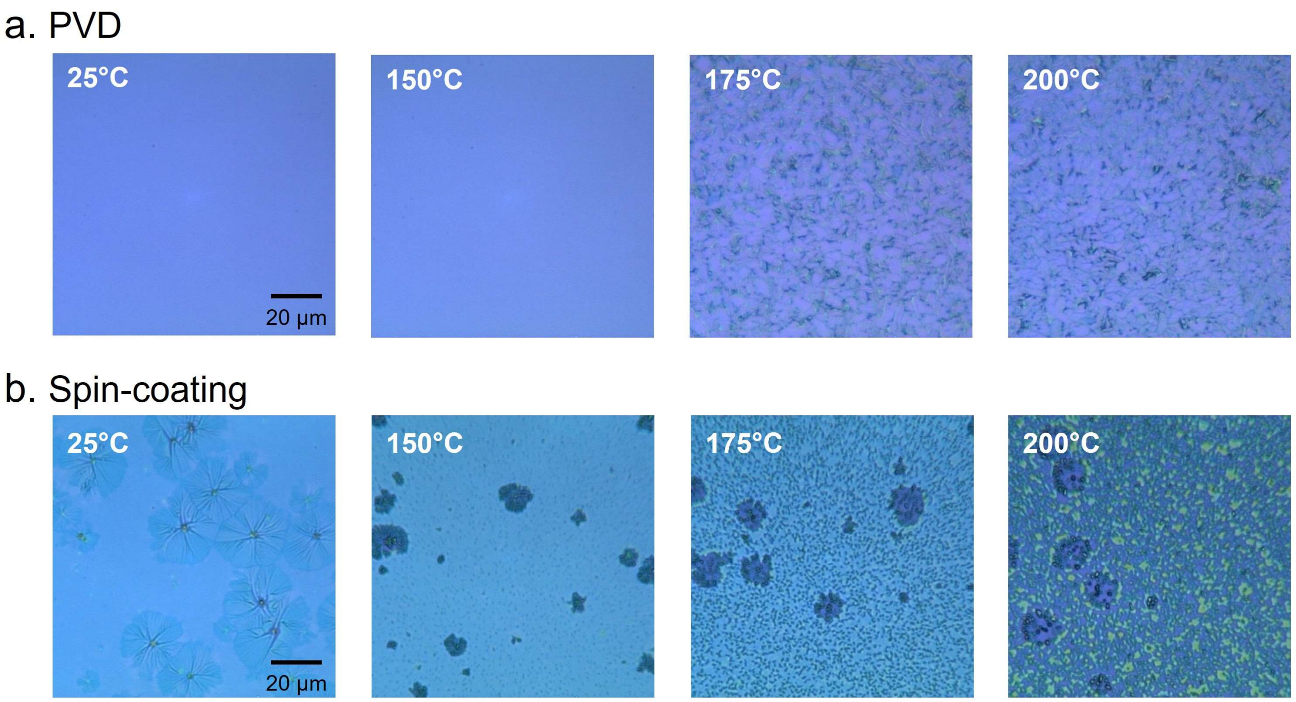

Figure S4. Bright field real-colour microscopy images of $(3PS)_2$-SiPc thin-films fabricated by (a) PVD and (b) spin-coating thermally annealed 25ºC, 150ºC, 175ºC, and 200ºC.

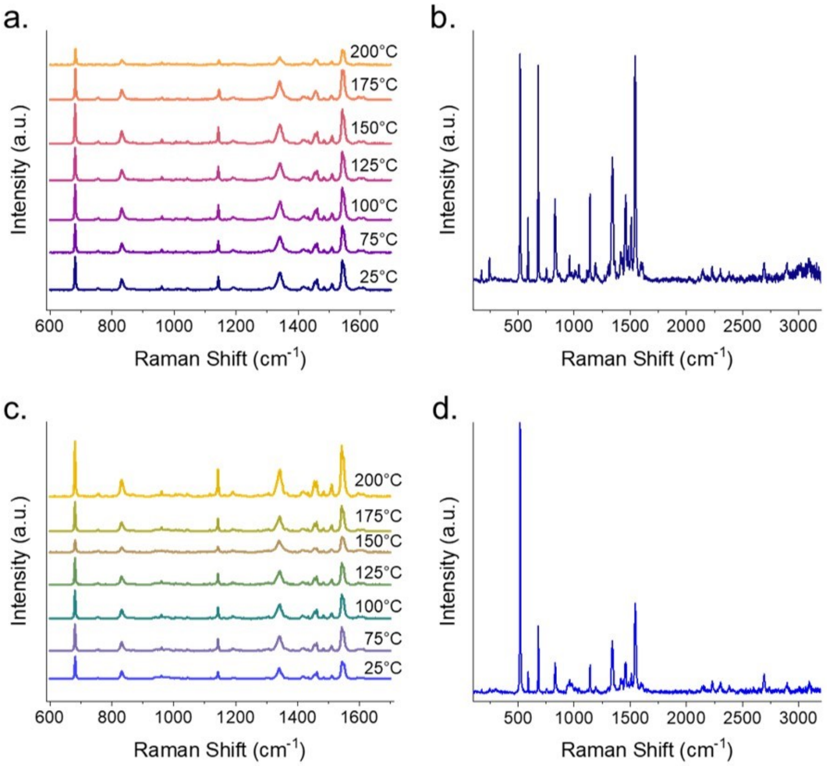

Figure S5. Raman spectra of thermally annealed $(3PS)_2$-SiPc thin-films fabricated by (a) PVD and (c) spin-coating. Extended Raman spectra of $(3PS)_2$-SiPc thin-films annealed at 25°C fabricated by (b) PVD and (d) spin-coating.

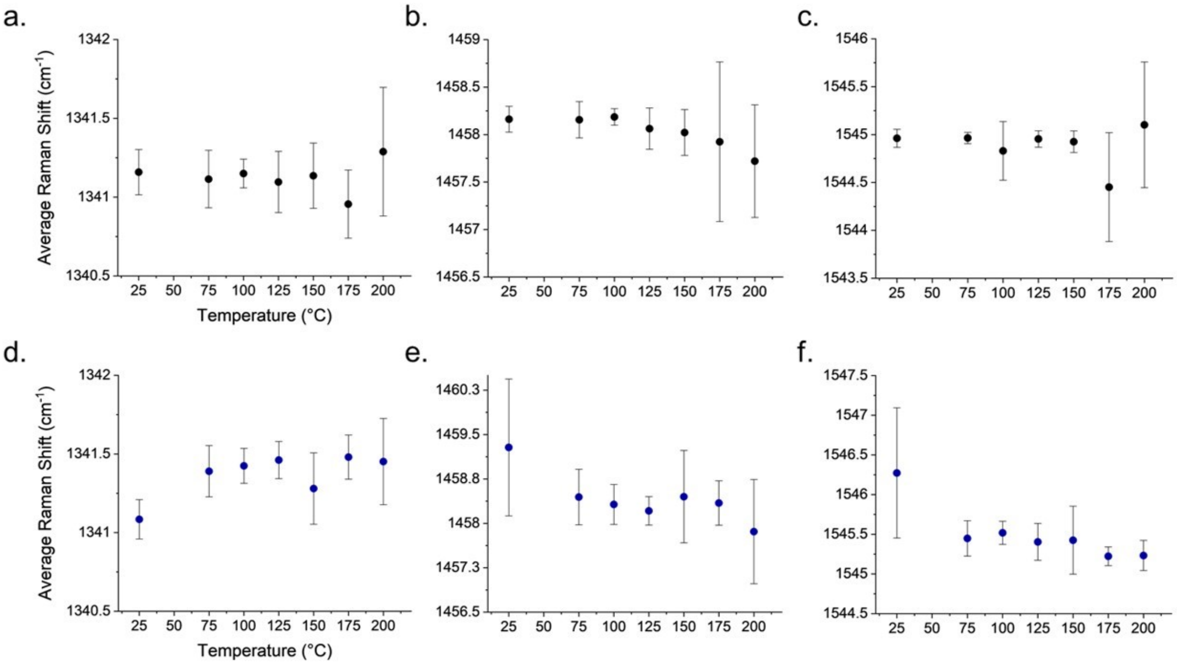

Figure S6. Average shift in (a,d) 1341 cm^{-1}, (b,e) 1458 cm^{-1}, and (c,f) 1545 cm^{-1} peak location with increasing annealing temperature observed in the Raman spectra of (a-c) PVD and (d-f) spin-coating fabricated $(3PS)_2$-SiPc thin-films. Averages taken from 10 individual measurements at each annealing temperature.

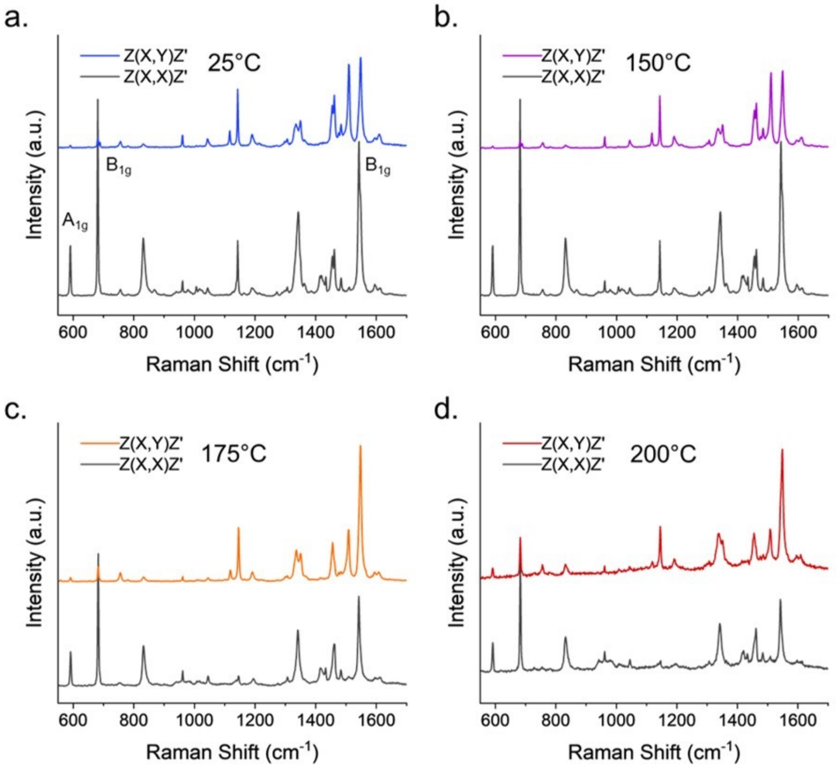

Figure S7. Polarized Raman spectra of $(3PS)_2$-SiPc thin-films fabricated by PVD thermally annealed at (a) 25°C, (b) 150°C, (c) 175°C, and (d) 200°C.

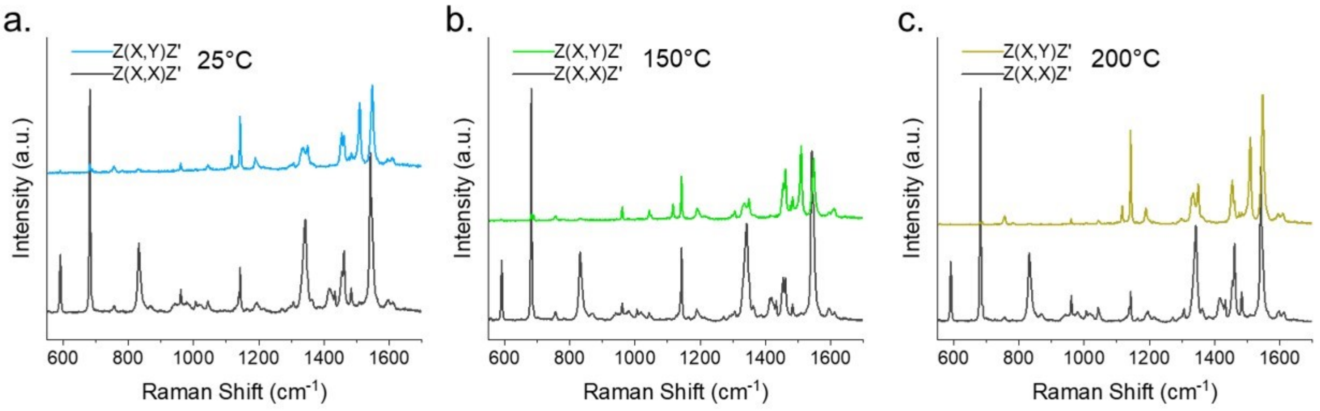

Figure S8. Polarized Raman spectra of $(3PS)_2$-SiPc thin-films fabricated by spin-coating thermally annealed at (a) 25°C, (b) 150°C, and (c) 200°C.

Table S2. Minimum, maximum and average molecular angle of $(3PS)_2$-SiPc to the substrate of PVD and spin-coated thin-films annealed at indicated temperatures, estimated from polarized Raman surface maps. Standard deviation (σ) calculated from the entire data set.

**Deposition**	**Temp.**	**β_{min}(degrees)**	**β_{max}(degrees)**	**β_{avg}(degrees)**	**σ(degrees)**
PVD	25°C	47.8	55.1	51.9	1.9
	150°C	47.0	51.3	49.2	0.7
	175°C	25.0	65.0	41.4	7.7
	200°C	30.6	73.6	56.6	10.4
Spin Coat	25°C	44.1	56.6	50.7	2.1
	150°C	43.3	55.4	50.4	2.1
	200°C	39.9	57.8	50.7	5.0

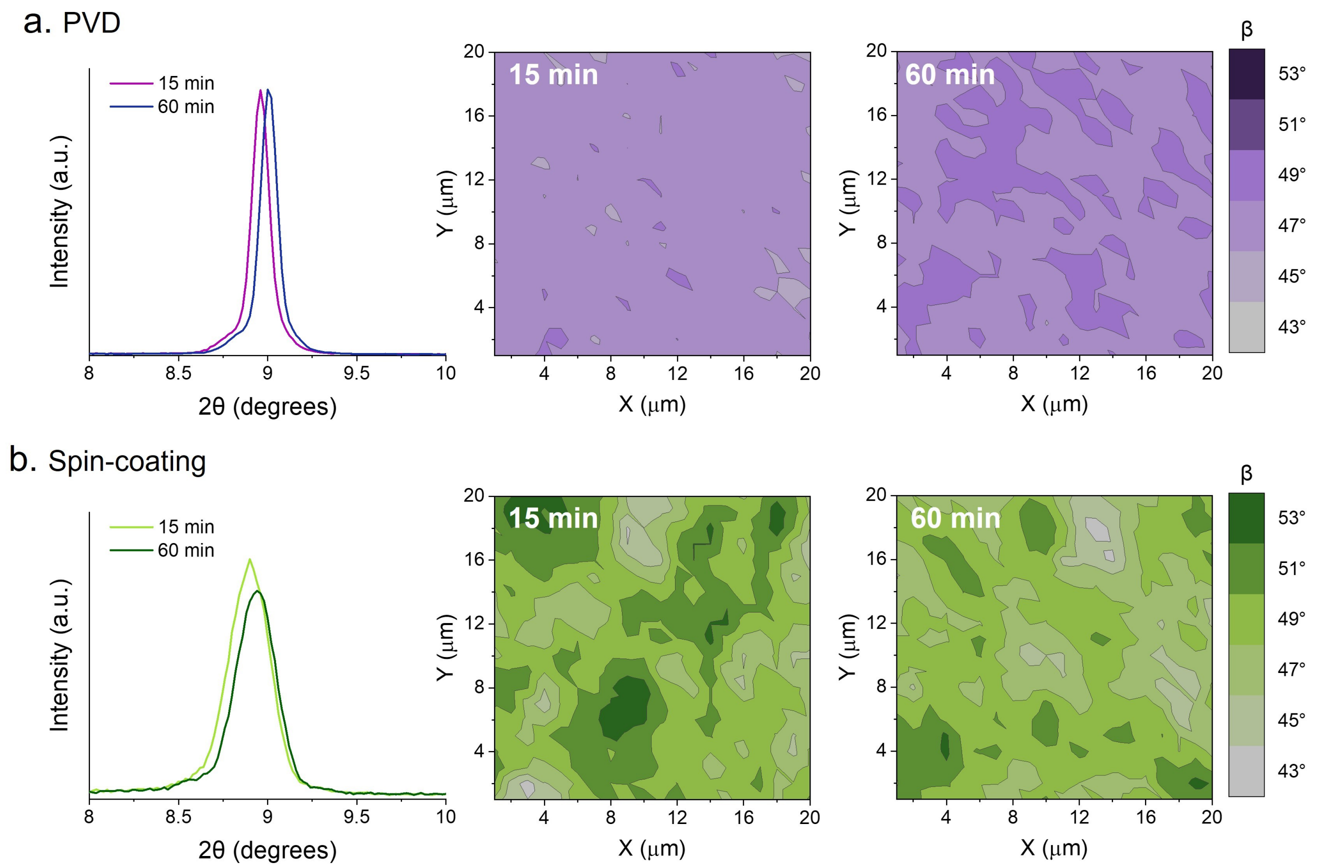

Figure S9. XRD patterns and maps (20 x 20 μm) of the molecular angle between $(3PS)_2$-SiPc and substrate estimated from polarized Raman spectra of $(3PS)_2$-SiPc thin-films fabricated by (a) PVD and (b) spin-coating thermally annealed at 100°C for 15 min and 60 min.

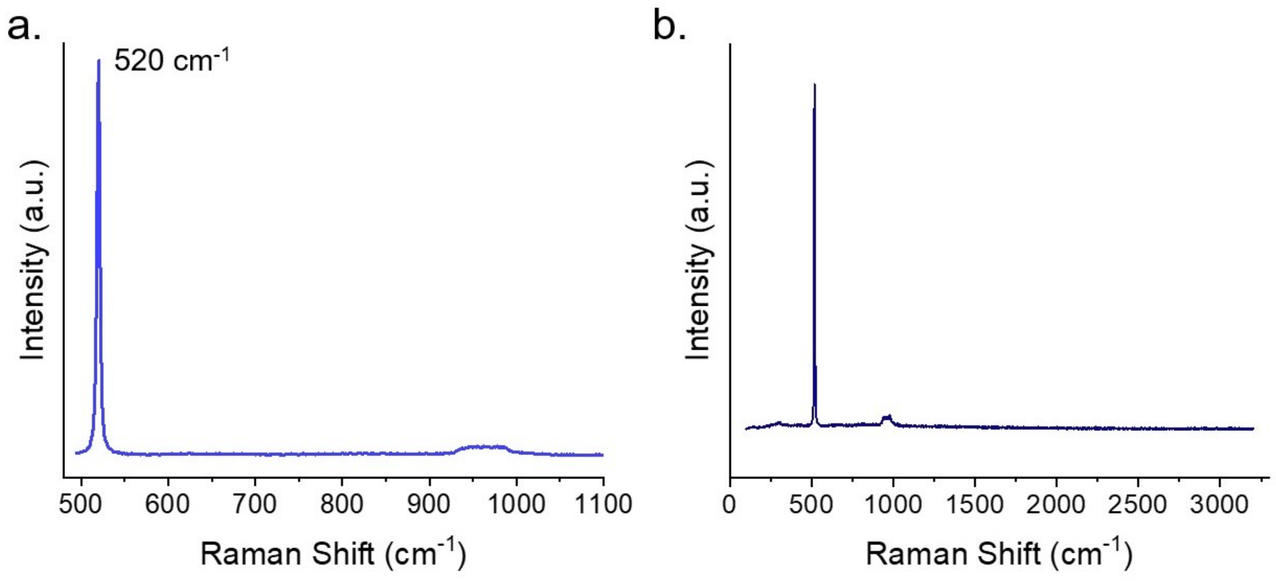

Figure S10 . Raman spectra of a clean SiO_2substrate from (a) 495-1685 $cm^{-1}$and (b) 100-3200 cm^{-1}.

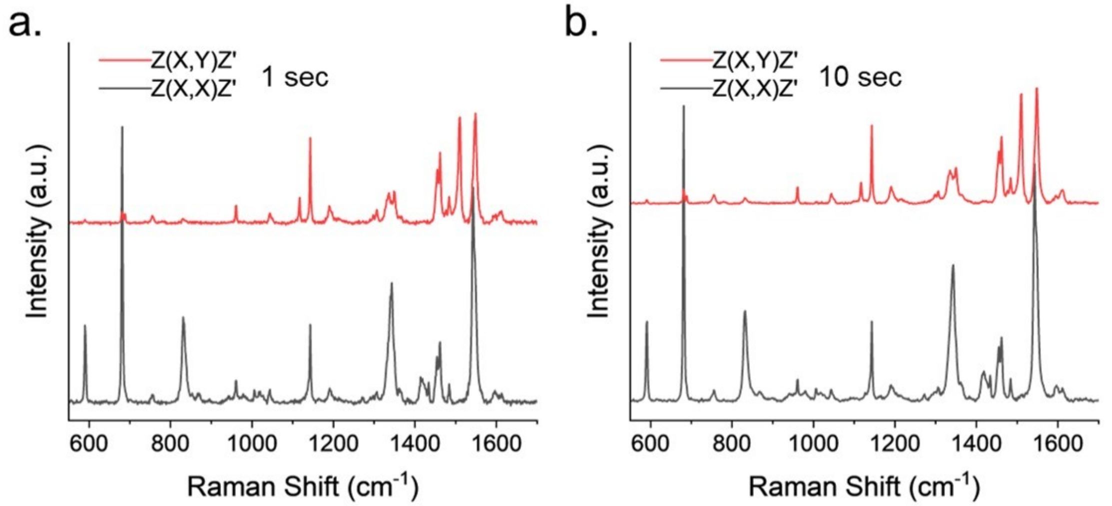

Figure S11 . Polarized Raman spectra of $(3PS)_2$-SiPc thin-films fabricated by PVD thermally annealed at 25°C, with (a) 1 sec and (b) 10 sec exposures to the laser during measurement.

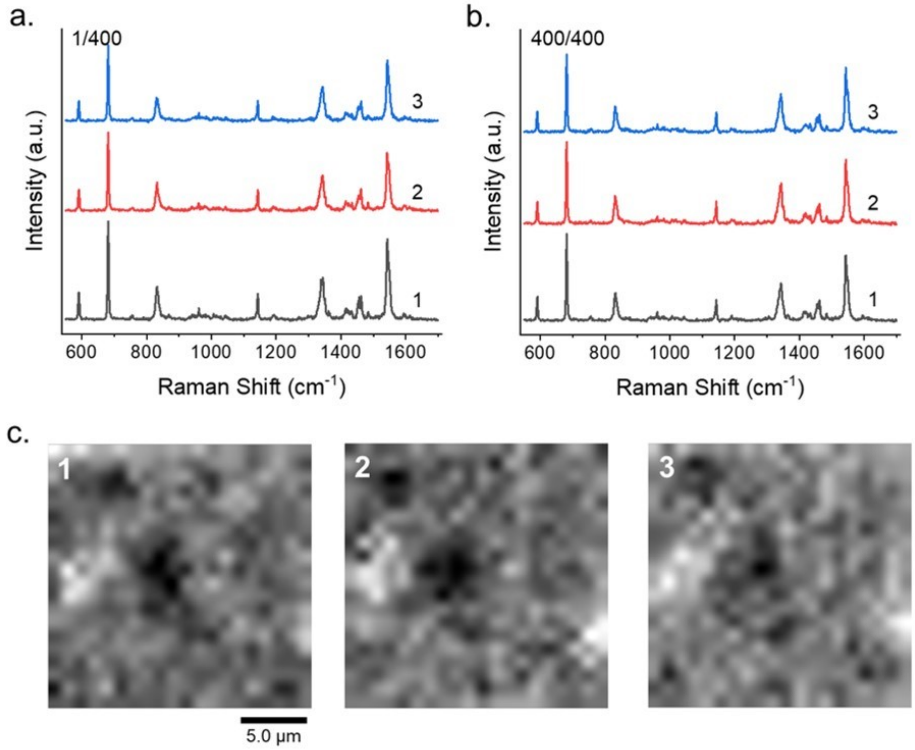

Figure S12 .Z(X,X)Z' polarized Raman spectra taken from three sequential Raman maps of $(3PS)_2$-SiPc thin-films fabricated by PVD thermally annealed at 25°C, at (a) the start of scan (1/400 measurements) and (b) end of scan (400/400 measurements). (c) Sequential intensity at B_{1g} 1545 cm^{-1} maps (20 x 20 µm) of films.

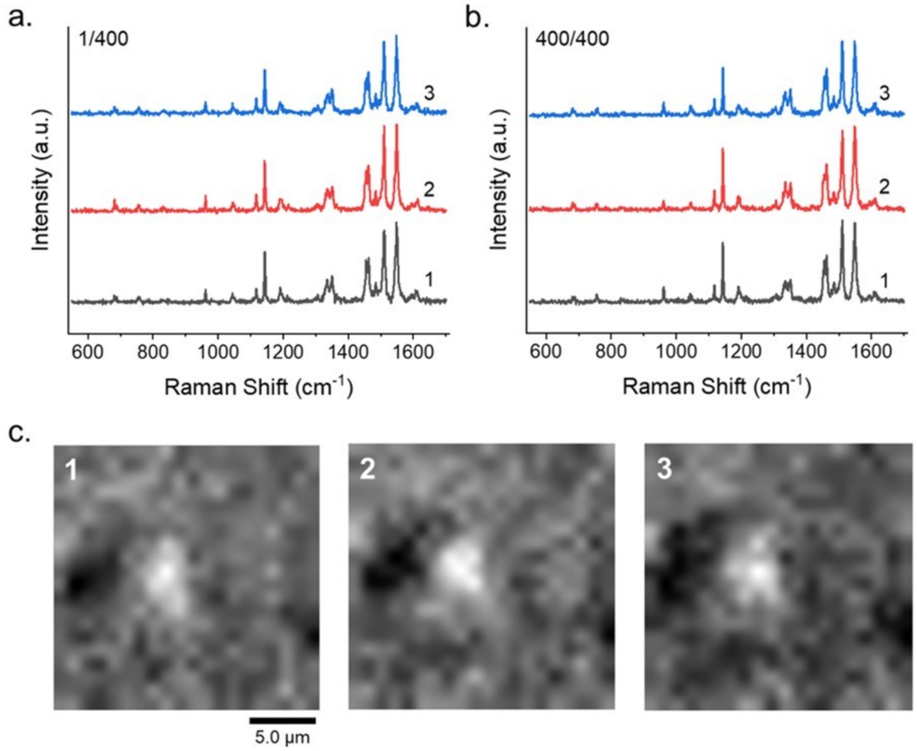

Figure S13 . Z(X,Y)Z’ polarized Raman spectra taken from three sequential Raman maps of $(3PS)_2$-SiPc thin-films fabricated by PVD thermally annealed at 25°C, at (a) the start of scan (1/400 measurements) and (b) end of scan (400/400 measurements). (c) Sequential intensity at B_{1g} 1545 cm^{-1} maps (20 x 20 µm) of films.

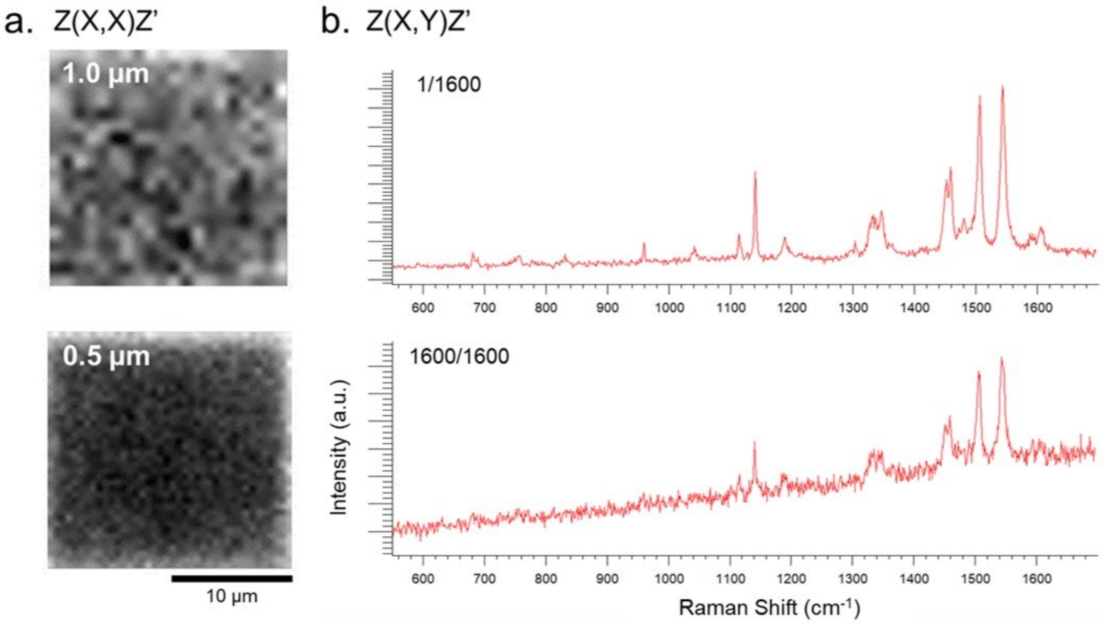

Figure S14 .(a) Comparison of intensity at B_{1g} 1545 cm^{-1} maps (20 x 20 µm) of $(3PS)_2$-SiPc films using a step size of 1.0 µmand 0.5 µm with Z(X,X)Z’ polarization. (b) Z(X,Y)Z’ polarized Raman spectra taken from intensity at B_{1g} 1548 cm^{-1} Raman maps using a step size of 0.5 µm, at the start of scan (1/1600 measurements) and end of scan (1600/1600 measurements).
